# Infection in the Developing Brain: The Role of Unique Systemic Immune Vulnerabilities

**DOI:** 10.3389/fneur.2021.805643

**Published:** 2022-01-24

**Authors:** Gabriela Singh, Elizabeth W. Tucker, Ursula K. Rohlwink

**Affiliations:** ^1^Division of Neurosurgery, Department of Surgery, Neuroscience Institute, University of Cape Town, Cape Town, South Africa; ^2^Department of Anesthesiology and Critical Care Medicine, Johns Hopkins University School of Medicine, Baltimore, MD, United States; ^3^Francis Crick Institute, London, United Kingdom

**Keywords:** central nervous system (CNS) infections, vulnerabilities, developing brain, immune response, children, peripheral immune system

## Abstract

Central nervous system (CNS) infections remain a major burden of pediatric disease associated with significant long-term morbidity due to injury to the developing brain. Children are susceptible to various etiologies of CNS infection partly because of vulnerabilities in their peripheral immune system. Young children are known to have reduced numbers and functionality of innate and adaptive immune cells, poorer production of immune mediators, impaired responses to inflammatory stimuli and depressed antibody activity in comparison to adults. This has implications not only for their response to pathogen invasion, but also for the development of appropriate vaccines and vaccination strategies. Further, pediatric immune characteristics evolve across the span of childhood into adolescence as their broader physiological and hormonal landscape develop. In addition to intrinsic vulnerabilities, children are subject to external factors that impact their susceptibility to infections, including maternal immunity and exposure, and nutrition. In this review we summarize the current evidence for immune characteristics across childhood that render children at risk for CNS infection and introduce the link with the CNS through the modulatory role that the brain has on the immune response. This manuscript lays the foundation from which we explore the specifics of infection and inflammation within the CNS and the consequences to the maturing brain in part two of this review series.

## Introduction

Central nervous system (CNS) infections in children continue to be an important cause of significant morbidity and mortality, with a predominance in low- and middle-income countries (1–3). To unravel the complexity of these infections in children it is important to consider the peripheral (immune system outside of the CNS) and central (immune system within the CNS) immune systems, as well as neuro-physiological and neuro-anatomical factors that may add to pediatric vulnerability or resilience.

The development of the human immune system is a continuous process and undergoes various changes during infancy and childhood, potentially gaining peak function during adolescence which carries over into adulthood. There are various defense barriers that form part of host immunity. The skin and mucosal membranes comprise the physical defense barrier. They undergo developmental, functional and structural changes with age, which are influenced by appropriate microbial exposure postnatally [extensively reviewed by (4)]. On a cellular level, the pediatric immune system comprises mostly naïve cell populations which similarly undergo age-specific maturation that is influenced by environmental stimuli. Consequently, young children are at greater risk of developing infections compared to older children and adults, and are more vulnerable to disseminated infections, including infections of the CNS. For instance, rates of meningitis secondary to *Hemophilus influenzae, Streptococcus pneumonia* and *Mycobacterium tuberculosis* are highest among children <5 years of age, or in adults with compromised immune systems secondary to human immunodeficiency virus (HIV) (5–8). Across 5 countries of the meningitis belt in Africa, ~74% of bacterial meningitis cases between 2015 and 2017 occurred in children under the age of 14 years (9). Further, a study comparing adult and pediatric tuberculous meningitis (TBM) found a significantly higher rate of miliary TB in children, likely indicative of the immature immune system's reduced capacity to contain the infection and prevent dissemination (10). Herpes simplex encephalitis is also known to be more severe in children compared to adults given lower neonatal production of type 1 interferon and impaired autophagy (11). Other diseases and infections characterized by inflammation and active immune responses, such as juvenile-onset systemic lupus erythematosus (12), Behçet's disease (13), para-infectious optic neuritis (14), and severe malaria (15), also report different clinical manifestations in children compared to adults.

Additionally, neurodevelopment may influence the impact that CNS infections have on the developing brain. For instance, age-related changes are seen in brain metabolism, which is dependent on myelination and synaptogenesis, cerebral blood flow, the balance between intracranial CSF and brain tissue, and skull development, such as the closure of fontanels and sutures. These factors have significant implications for children with regards to their vulnerability to brain and/or spinal injury and the consequences thereof (16).

Therefore, children cannot simply be considered “little adults,” but rather warrant pediatric-specific treatment strategies that are tailored to their unique developmental physiology. In this review, we summarize the current evidence for peripheral immune characteristics across childhood that render children at risk for CNS infection and introduce the bidirectional link with the CNS through the modulatory role that the brain has on the immune response. Although we use data available from human studies whenever possible, we have also utilized *in vitro* and *in vivo* animal studies which also provide valuable data. For the purpose of this review we have established the age categories as follows: neonate (infants in the first 28 days after birth), infants (0–1 years), preschool children (1–6 years), primary school children (6–12 years), adolescents (12–18 years) and adults (>18 years); unless otherwise stated as specific to the study referenced. This manuscript lays the foundation from which we explore the specifics of infection and inflammation within the CNS and the consequences to the maturing brain in part two of this review series.

## Peripheral Immune System

**Figure 1:**
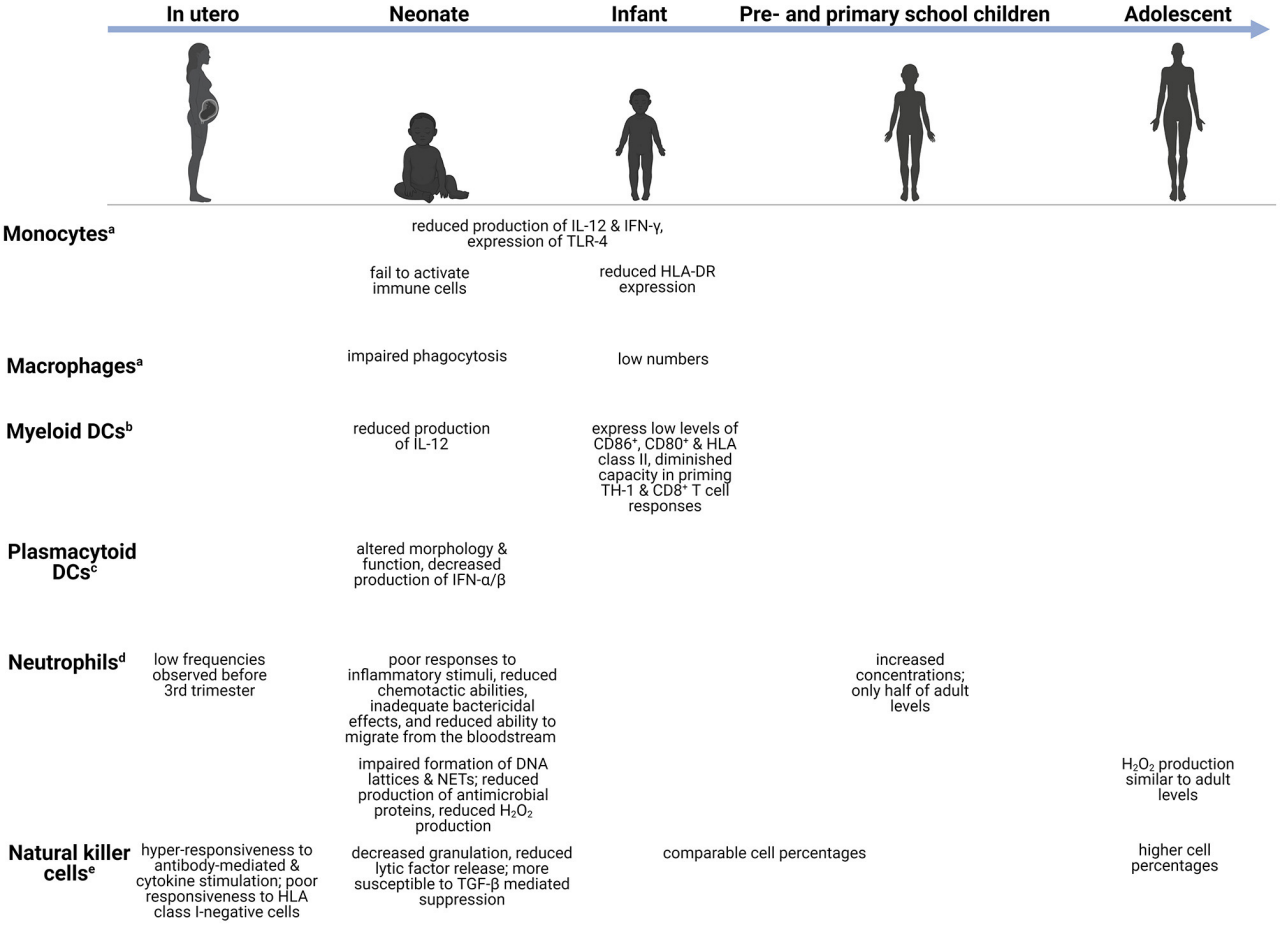
Age-dependent differences in the innate immune system. Changes in the innate immune cells during development, from *in utero* (week 27–birth), neonate (infants in the first 28 days after birth), infants (0–1 years), preschool children (1–6 years), primary school children (6–12 years), to adolescents (12–18 years). ^a^Monocytes/macrophages-capable of phagocytoses and production of pro-inflammatory cytokines (18). ^b^Myeloid DCs-potent initiators of TH1-mediated responses (19). ^c^Plasmacytoid DCs-act as antigen presenting cells and regulate T cell responses (19). ^d^Neutrophils-release antimicrobial peptides and produce reactive oxygen species (20, 21). ^e^Natural killer cells-release granzyme B and perforin (22). CD, cluster of differentiation; DC, dendritic cell; DNA, deoxyribonucleic acid; HLA-DR, human leukocyte antigen-DR; H_2_O_2_, hydrogen peroxide; IL, interleukin; IFN, interferon; NET, neutrophil extracellular trap; TGF-β, transforming growth factor β; TH, T-helper; TLR, toll-like receptor.

**Figure 2:**
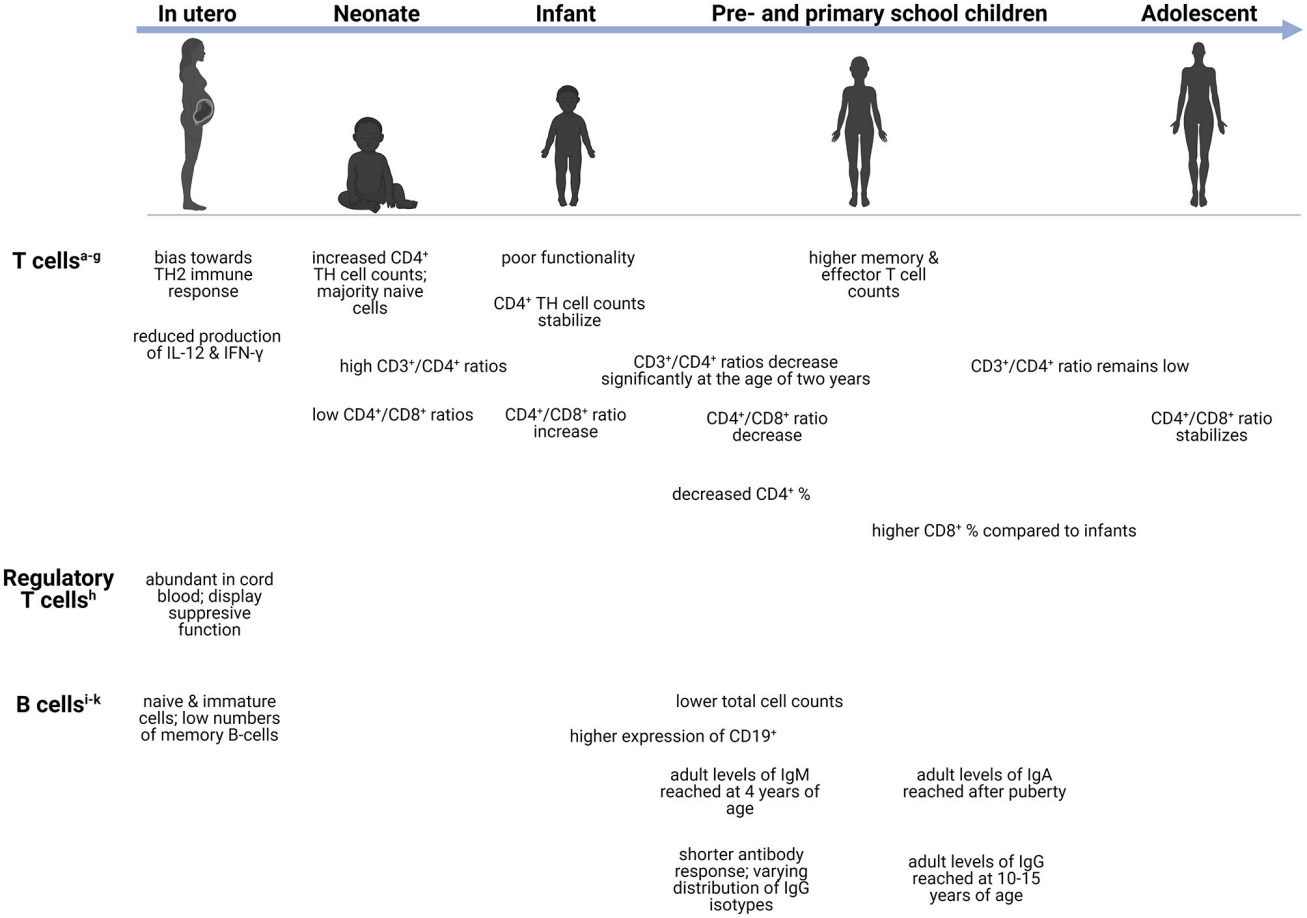
Age-dependent differences in the adaptive immune system. Changes in the adaptive immune cells during development, from neonate (infants in the first 28 days after birth), infants (0–1 years), preschool children (1–6 years), primary school children (6–12 years), to adolescents (12–18 years). ^a^CD4^+^ T cells-helper cells that produce variety of cytokines (23). ^b^TH1 cells-produces IFN-γ and initiates protective immune responses against viral/bacterial infections (24). ^c^TH2 cells-produces IL-4, IL-5, IL-9, IL-13, and IL-15 (24). ^d^TH9 cells-produces IL-9, IL-10, and IL-21 (25). ^e^TH17 cells-respond to extracellular bacteria and fungi (24). ^f^CD3^+^ T cells-important for activation of CD4^+^ and CD8^+^ T cells (26). ^g^CD8+ T cells-cytotoxic cells capable of TNF-α and IFN-γ mediated killing (27). ^h^Treg cells-immunosuppression of CD4^+^ and CD8^+^ cell function; release inhibitory cytokines (24). ^i^Memory B cells-exerts immunosuppression; can rapidly differentiate into effector cells following antigen recognition (28). ^j^Transitional B cells-exerts immunosuppression through IL-10 production (29). ^k^Plasma blasts-most mature class-switched subset of memory B cells (30). CD, cluster of differentiation; Ig, immunoglobulin; IL, interleukin; IFN-γ, interferon-γ; TH, T-helper; TNF-α, Tumor necrosis factor-α.

The immune system is equipped with a vast array of cells and immune modulators capable of sensing internal and external stimuli, initiating host defense against pathogens, and maintaining tissue homeostasis (17). Distinct immune features are present during each life stage (Figures 1, 2), introducing unique age-related challenges that may impact the response to infections and increase children's vulnerability to CNS infections.

### Innate Immune System

The innate immune system forms the first line of defense against invading pathogens, comprising monocytes, macrophages, dendritic cells (DCs), neutrophils, and natural killer (NK) cells (17). These innate immune cells share the ability to rapidly detect pathogen-associated molecular patterns (PAMPs) and danger-associated molecular patterns (DAMPs) through a limited number of germ-line encoded pattern-recognition receptors (PRRs). Activation of PRRs *via* non-sterile damage (e.g., microorganisms or cytoplasmic PAMPs) or sterile damage (e.g., neurotoxins, chemical compounds, endogenous DAMPs) stimulates intracellular signaling that leads to inflammasome mobilization and thereby caspase-1 activation, proteolysis, release of interleukin (IL)-1β and IL-18, NF-κB activation, and pyroptotic cell death (31–34). Members of the PRR family involved with innate immunity include Toll-like receptors (TLRs), nucleotide-binding oligomerization domain (NOD)-like receptors (NLRs), retinoid acid-inducible gene-1-like receptors (RIG1), and C-type lectin receptors (34). The TLR family (35) consists of cell surface-expressed TLRs (TLR1, TLR2, TLR4, TLR5, TLR6, and TLR11) that detect microbial structures such as bacterial and fungal cell wall components along with viral proteins; intracellularly-expressed TLRs (TLR3, TLR7, TLR8, and TLR9), in turn, function to detect RNA of foreign origin (36–40). Numerous studies demonstrate attenuation of TLR responses in young children with maturation of the TLR pathways occurring across childhood. This may in turn contribute to the developmental profile of cytokine responses with age (41).

Development of innate immune responses begins during the fetal period, progresses during early childhood at various cell-specific rates, and reaches full capacity during adolescence (42). Monocytes and macrophages serve as significant effector cells of the innate immune system by carrying out phagocytic functions, producing key cytokines, and presenting phagocytosed microorganisms to T lymphocytes (T cells) (41). Quantitatively, these cell populations are often decreased in young children. For example, pulmonary macrophages are low in preterm and term infants but reach adult cell levels days following birth (43). Although numbers may increase, important differences in functionality often remain. Monocytes and macrophages exhibit reduced production of cytokines such as IL-12 and interferon (IFN)-γ, and reduced TLR-4 expression in neonates and preterm infants compared to adults (17, 44). In addition to cytokine production, phagocytosis of pathogens and subsequent intracellular bacterial killing are important components of the innate immune system. Several reports observed impaired phagocytic functions by neonatal monocytes, macrophages, and neutrophils (45–48). The impairment appeared to be transient, as adult levels of neonatal phagocytic abilities are reached 3 days following birth, however, the impact thereof on neonatal bacterial susceptibility remains unexplored (48, 49). Additionally, neonatal monocytes fail to activate adaptive immune cells, either through reduced human leukocyte antigen (HLA)-DR expression or inadequate cytokine production (48). In a separate study, reduced HLA-DR expression by monocytes was suggested to play a part in the increased risk of developing infectious complications in preterm infants (50).

Similar to monocytes/macrophages, DCs serve as a vital link between innate and adaptive immunity by processing and presenting antigens to adaptive immune cells. Myeloid DCs (mDC) produce low levels of IL-12 and are few in numbers early on in life. Low expression of CD86^+^, CD80^+^ and HLA class II, as well as a diminished capacity in priming CD4^+^ T helper (TH) 1 and CD8^+^ T cell responses, have also been noted in mDCs in young children (51). Concomitantly, these impairments correlate with children's susceptibility to disseminated infections such as *Salmonella* spp and *M. tuberculosis* (52). Interesting, plasmacytoid DCs (pDC) show a greater degree of impairment (53). Schuller et al. observed that pDCs of preterm neonates displayed altered morphology and cell function, with significant decreases in the production of IFN-α/β in response to different viral infections (54). These failures to activate the adaptive immune system leave young children vulnerable to infection.

Neutrophils are the most abundant polymorphonuclear cells and along with monocytes/macrophages, form the phagocytic system of the innate immune system (48). Low numbers of neutrophils are observed just before the third trimester but increase significantly shortly before birth. Preschool children have increased neutrophil concentrations compared to infants, but these concentrations are still only half of adult levels (55). Although the number of circulating neutrophils is high in the days following birth, they demonstrate poor responses to inflammatory stimuli, reduced chemotactic abilities, inadequate bactericidal effects, and reduced ability to infiltrate into the tissues from the bloodstream (48, 56). Neutrophil infiltration into the CNS is a hallmark of bacterial meningitis and an important bacterial meningitis diagnostic criterion (57). Therefore, failure of children's neutrophils to infiltrate into the CNS may contribute to their increased risk of CNS infections. Furthermore, neutrophils' ability to produce hydrogen peroxide (H_2_O_2_), one of the reactive oxygen species (ROS), in response to *Escherichia coli* and *Staphylococcus aureus*, is significantly reduced in infants. The level of H_2_O_2_ production gradually increases with age, as children aged 10–15 years have similar production levels to adults (58). In addition to these functional deficiencies, Yost et al. reported impaired formation of DNA lattices and neutrophil extracellular traps (NETs) as well as reduced production of antimicrobial proteins in neonates, which contribute to decreased extracellular bacterial killing (59). Impaired tissue infiltration and microbial killing by neutrophils are crucial risk factors for uncontrolled infections.

The lymphoid cell line of innate immunity comprises NK cells and innate lymphoid cells (ILCs) consisting of distinct populations: group 1 ILCs, group 2 ILCs, and group 3 ILCs (60). NK cells can identify and kill virus-infected cells *via* cytotoxic mechanisms and IFN-γ production without prior sensitization (61). There are two subpopulations of NK cells which can be distinguished by the cell surface density of CD56^+^, differing both functionally and phenotypically. The CD56^bright^ subset serves as the primary source of NK cell-derived immunoregulatory cytokines, whereas CD56^dim^ mediates natural cytotoxicity (61). Animal models of bacterial meningitis and cerebral malaria have found significant infiltration of NK cells secreting IFN-γ (62). In fetal NK cells, Ivarsson et al. observed hyper-responsiveness to both antibody-mediated and cytokine stimulation, and poor responsiveness to HLA class I-negative cells. Additionally, NK cell release of lytic factors and degranulation are decreased during early life compared to older individuals (63). Several studies have also reported neonatal NK cells to be more susceptible to transforming growth factor (TGF)-β mediated suppression than adults, suggesting that this multifunctional cytokine contributes to NK cell immaturity during early development (64, 65). Infants and preschool children have comparable NK cell percentages, whereas adolescents possess significantly higher percentages (55). This is in keeping with findings of previous studies reporting an increase in the percentage rather than the number of NK cells with age (66, 67). ILCs can produce a wide variety of cytokines and participate in immunity against intra- and extracellular bacteria, metabolic homeostasis, and lymphoid tissue development (60). High frequencies and absolute numbers of ILCs are present in cord blood and peripheral blood of primary school children and adolescents, whereas decreased frequencies of group 2 and 3 ILCs are observed in adults (68).

Although considered part of the innate immunity, the complement system serves multi-functional roles in both innate and adaptive immunity. These include initiating proinflammatory responses through the close interaction with TLRs, NLRs and inflammasomes, participating in B lymphocytes (B cells) and T-cell responses, and cell homeostasis (69). The activity of the complement system is subdued during early life compared to adults (4). In a study which reviewed a spectrum of primary immunodeficiencies at a tertiary pediatric hospital in South Africa, more than half of the children (median age 10 years) with complement deficiencies, particularly in C3 and C6, experienced recurrent meningococcal meningitis (70). Furthermore, complement proteins are also produced by resident cells of the CNS, and appear to play a vital role in the immunity against Herpes simplex encephalitis (69, 71).

Although several innate immune cells reach adult levels early in childhood, the majority lack full functionality, with impaired responses to inflammatory stimuli, inadequate production of cytokines and poor microbial killing. Consequently, young children are more susceptible to infections, some of which can disseminate to the CNS.

### Adaptive Immune System

The adaptive immune response commences when innate responses are unable to successfully eliminate the pathogen and requires antigen presentation by innate immune cells. Adaptive immunity develops due to prior exposure to a stimulus and can be characterized as cell-mediated or antibody-mediated response (72, 73). Although we highlight the distinction between cell-mediated and antibody-mediated response below, both T cells and (B cells) are needed for an appropriate antibody-mediated response as the T cells, specifically CD4^+^ TH cells, are needed for antigen presentation, B cell activation, and antibody production.

#### Cell-Mediated Immunity

Conventional T cells can be divided into two functionally different types namely, CD4^+^ TH cells and cytotoxic CD8^+^ T cells (73). The former are responsible for prompting the function of other cells *via* excreted cytokines and can further be divided into regulatory and effector cells (TH1, 2, 9 and 17) (73, 74). At birth, T cells are increased and remain so throughout early infancy and normalize to adult levels in adolescence (66, 73). Despite these high levels, the functionality of these cells is poor, with diminished fetal IL-12 production, which plays a crucial role in T cell functionality (74). Interestingly, both term and preterm neonates demonstrate increased CD4^+^ TH cell counts after birth that quickly stabilize during infancy; however, most are naïve cells (73). On the contrary, significant decreases in CD4^+^ percentages are observed throughout infancy, childhood, and adolescence, only increasing significantly during adulthood (55). The ratio of CD3^+^/CD4^+^ T cells remain high from birth until infancy and decrease significantly after 2 years of age, remaining low throughout early childhood, adolescence, and adulthood (66). Functional CD4^+^ T cells are important during bacterial meningitis secondary to *Hemophilus influenzae, Streptococcus pneumoniae*, and *Neisseria meningitidis* (75). Children with these bacterial meningitides demonstrated increased CD4^+^ T cells with elevated CD4^+^ CD45R+ (suppressor-inducer T cells) and depressed CD4^+^ CDw29^+^ (helper-inducer, or memory T cells) but decreased CD8^+^ T cells, suspected to correlate with impaired antibody responses (75). In tuberculosis (TB), a lower abundance of genes responsible for T-cell activation, proliferation, and receptor signaling, as well as reduced peripheral T-cell responses, are more profoundly seen in children with TBM as opposed to those with pulmonary TB, suggesting that these impairments may play a role in dissemination of the disease to the brain (76, 77). Similar to several innate immune cells, T cells are often depleted in number or, if normal to high levels, are dysfunctional with low cytokine production which can limit effective adaptive immune response.

The developing fetus exhibits an immune response skewed toward TH2 immunity, which is characterized by the reduced production of IFN-γ by T cells. Regulatory T (Treg) cells are abundant in cord blood and display a suppressive function on immune responses (78, 79). Treg cells along with resident CNS immune cells help regulate neuroinflammation following CNS infection by suppressing proinflammatory mediators (79). Given the early recruitment of peripheral effector cells during CNS infection, the timing of Treg-mediated immune responses is crucial in ensuring resolution of neuroinflammatory processes and minimizing brain injury associated with excessive inflammation (80). For example, recruitment of increased Treg cells during the early phase of West Nile Virus infection has been associated with improved patient outcome (80). Furthermore, in a viral encephalomyelitis model, depletion of Treg cells in a diphtheria toxin-dependent manner resulted in severe CNS inflammation and subsequent neuronal damage despite no impact on viral clearance (81). Increased frequencies of Treg cells were also observed in children with various forms of extra-pulmonary TB disease including TBM, which persisted even following treatment (82). This could potentially reflect an underlying immune susceptibility to disseminated TB disease.

Contrary to CD4^+^ TH cells, CD8^+^ T cells exert cytotoxic function by killing infected cells (74, 83). Pre-school children, adolescents and adults, exhibit significantly higher CD8^+^ T cell percentages compared to infants (55). Risdon et al. demonstrated that neonatal CD8^+^ T cells lacked appropriate responses and required a greater stimulus to elicit their function (83). Moreover, CD4^+^ and CD8^+^ T cells can also acquire a memory phenotype following infection; however, low numbers are present in healthy neonates and remain low even in older children (83). Compared to neonates, adults and children (aged 5–10 years) possess higher counts of memory and effector T cells (73). The ratio of CD4^+^/CD8^+^ T cells also exhibits fluctuation with age: low values are observed at birth, followed by increases during the first 2 years of life, a decrease throughout childhood, and finally stabilizing during adulthood (66). Both CD4^+^ and CD8^+^ T cells participate in pathogen clearance following CNS infection. Increased levels of CD4^+^ T cells were found in the CSF in some forms of meningitis during acute infection (84). Furthermore, CD4^+^ TH cells are important in maintaining CD8^+^ T cell-mediated responses during CNS infection. Gradual CD4^+^ TH cell depletion in a mouse viral encephalitis model affected infiltrating CD8^+^ T cell effector functions and led to impaired viral control (85). Specifically, IFN-γ production and granzyme B expression were significantly reduced, however CD8^+^ T cell recruitment to the CNS was only slightly diminished (85). Functional exhaustion of T cells can be induced during chronic infections, and although observed in both adults and infants, the latter group experiences greater exhaustion, as reported during congenital cytomegalovirus (CMV) infection (86). These alterations in T cell levels and functionality could have significant implications in the pathogenesis of CNS infections during early life.

#### Antibody-Mediated Immunity

B cells are the driving force behind the antibody-mediated branch of the adaptive immune system by secreting antibodies. The immunoglobulin (Ig) molecules, or antibodies, exist in four different isotypes in humans, namely IgM, IgA, IgG and IgE. Once activated, B cells undergo isotype switching which allows them to subsequently secrete antibodies of the different isotypes. Isotype switching involves altering the effector function of the secreted antibody without influencing antibody specificity (87).

Throughout life, peripheral B cells differentiate and mature when confronted with foreign antigens; however, the most discernible changes in the composition of the B cell pool occur during the first 5 years of life (88). Resultantly, infants rely heavily on their innate immune system for protection against infections given the reduced ability to mount appropriate and prompt adaptive responses due to lack of antigen exposure *in utero* (89). Maternally derived antibodies exert their influence during the neonatal period by shaping the B cell repertoire, allowing for enhanced mucosal immunity and antimicrobial protection (90, 91). The number of B cells has been shown to decrease with age as children (5–10 years old) and adults exhibit lower levels compared to neonates (73, 88). The expression of CD19^+^, an antigen found on all B cells that is also used as a biomarker of B cell development, was found to be highest in infants and young children compared to older age groups (55). Flow cytometric data have shown that the majority of the B cells at birth are naïve and immature, with few memory B cells, reflecting a lack of antigenic stimulation (73). The percentage of naïve B cells as well as transitional B cells (CD24^++^CD38^++^) decreases significantly with age (88). Interestingly, a mouse model of pneumococcal meningitis showed that both T cells and B cells were needed to delay spread to the CNS and improve survival (92), demonstrating the importance of functional T cells and B cells to prevent CNS dissemination.

Age-related changes have also been observed with respect to antibody production. The number of switched and non-switched IgG isotype memory B cells increases slowly during childhood with adult numbers only being reached by 10–15 years of age (88, 93, 94). Adult levels of IgA are only reached after puberty, whereas adult levels of IgM are reached by 4 years of age (94). Compared to adults, infants also have a shorter antibody response duration and varying distribution of IgG isotypes (93). Antibody-mediated responses are critical for the defense against viral CNS infections. More specifically, B cell- and antibody-deficient mice showed greater degree of dissemination to CNS in a West Nile virus infection model (95, 96). Similar to CD8^+^ T cells, B cell effector functions are also maintained by CD4^+^ TH cells' production of IL-21, reiterating the important interaction between T cells and B cells for normal immune function (96, 97).

As previously mentioned, T cells and B cells have an interactive relationship so alterations in adaptive immunity often involves both cells. The study of deficiency of both B cells and T cells using Rag1–/– mice demonstrated the importance of both cell types in delaying progression of pneumococcal meningitis (92). In this study, Ribes et al. showed that intracerebral *S. pneumoniae* normally induces B cell and T cell recruitment in wild-type mice. However, in Rag1–/– mice with no functional B cells or T cells, the mice had worse clinical symptoms and succumbed to the infection compared to wild-type mice (92).

Although data on the exact timing of immune vulnerabilities and development may be heterogenous, it is clear that distinct vulnerabilities are present in the pediatric innate and adaptive immune system. Peripheral immune cells have been shown to play an important role in local inflammatory responses during CNS infections, with a great body of evidence being derived from animal models and adult studies. However, while animal studies are incredibly valuable to understanding the mechanisms underlying health and disease, they are limited by the fact that animals' immune systems differ significantly from the human immune system. For instance, maturation of the immune system in rodent species (mice and rats), are delayed compared to humans [reviewed in greater detail by (98)]. Therefore, these developmental differences between animals and humans need to be considered when interpreting results from various species that are used to model the developing human immune system.

Given the immaturity of the developing immune system, young children are at a disadvantage when confronted with infections, not just at the level of the periphery but the CNS as well. However, further insight and research into the responses against CNS infections of children at different levels of maturity is required.

### Lymphatic System

The lymphatic system is an open-ended system capable of transporting fluid from the periphery back into circulation (99, 100). It plays an important role in immune cell differentiation, immune-surveillance and activation, immune cell trafficking, and facilitating antigen transport between the periphery and lymph nodes (101–103). Monocytes, DCs, neutrophils, T cells and B cells utilize lymphatic vessels for migration in both homeostasis and during infection (104–106). Chemokine receptor 7 (CCR7) appears to be the most universal regulator required for lymphatic migration among most immune cells. CCR7-deficient T cells, monocytes, and DCs demonstrate failed migration to popliteal/cervical lymph nodes and reduction in migration from the periphery in response to inflammatory stimuli, respectively (107–109). Moreover, the importance of CCR7 in immune cell migration to lymphatics was further emphasized in host defense, wherein CCR7-deficient mice exhibited increased susceptibility to both bacterial and viral infections (110–112). In neonates, CCR7 is downregulated which could have negative implications on their immune cell migration and host defense (113).

### Early Life Vaccinology

Given the limitations of the innate and adaptive immune responses in young children, establishing effective vaccination strategies are needed to confer immune protection within this vulnerable population and protect from disseminated infections (114, 115). Vaccines comprise the antigen-specific stimulus, known as immunogens, and the adjuvants, which are responsible for directing the quantity and quality of the elicited immune response (116, 117). Apart from improving vaccine efficacy in neonates, adjuvants enhance immunogenicity and reduce the number of immunizations required to establish protective immunity (118). Vaccinology in childhood remains challenging due to the continuous changes in immune responses during the first 5 years of life, ultimately representing a “moving” target of the optimal adjuvant due to age-dependent changes in adjuvant activity (117, 119, 120). Therefore, most vaccines administered in early life are subunit vaccines lacking adjuvant activity, which plays a crucial role in stimulating and shaping the immune response. There are several other challenges to effective immunization in children. Firstly, there are difficulties establishing the efficacy of vaccines given the restricted and short-lived antibody response in infants. The age of priming and at which the final dose in a series is given, along with dose intervals and vaccine type, are all factors affecting antibody responses to vaccines (121). A proposed way of circumventing the short-lived antibody responses is repeated administrations of the vaccine; however, this is often challenging to achieve in developing countries. Secondly, it is challenging to overcome the immunological milieu in neonates which is biased toward TH2 responsiveness. Thirdly, it is difficult to overcome the inhibitory effects of maternally derived antibodies which mask B cell epitopes; this inhibition is dependent on the ratio of maternally derived antibody titers and vaccine antigen dose (122). This inhibitory effect has been reported for most of the DNA vaccines as well as live and non-live vaccines. Despite these challenges surrounding immunization during childhood, certain vaccines, such as Bacille Calmette-Guerin (BCG) and Hepatitis B vaccine (HBV), have demonstrated safety and efficacy to a certain extent when given at birth (123), and the Hemophilus influenzae type b (Hib) vaccine dramatically reduced the incidence of Hib-associated meningitis in childhood (124).

### Factors Affecting Early Life Immunity

It is well established that young children are more prone to contracting infections due to their compromised immune system. Although immature, the developing immune system has the capacity to dynamically respond to the various influences. As discussed above, age-related changes within the developing peripheral immune system influence the response to infections and increase the risk of children to CNS infections. However, there are additional factors that may also have an effect. These factors may be intrinsic, like sex hormones, or environmental, such as malnutrition and maternal variables (i.e., maternal stress, malnutrition, etc.).

#### Sex-Related Immune Differences

The impact of sex hormones on the outcome of many infectious diseases, including invasive infections like meningitis, has been documented as early as infancy, with higher morbidity and mortality rates reported in males (125, 126). For instance, the male bias in bacterial infections has been observed in severe sepsis, *Hemophilus influenza, streptococcal* pharyngitis, *meningococcal* disease, and TB (127). Changes in sex hormones, which have a peak in infancy as well as during puberty, may contribute to the relative “honeymoon” of lower mortality and morbidity secondary to infectious diseases in school-aged children (4–14 years old) (128). Estradiol, testosterone, and progesterone have been shown to regulate macrophage, lymphocyte, and DC functionality. For instance, while testosterone exerts immunosuppressive effects by downregulating T cell-mediated IL-4 and IFN-γ production, estrogen enhances TH1 responses (129). Despite their tendency toward TH2 immunity, females are capable of mounting stronger cell- and antibody-mediated responses compared to their male counterparts (130, 131). Following the peak in sex hormones during adolescence, differences in the frequency of lymphocyte populations become apparent. For instance, males have a higher number of circulating B cells and NK cells in the periphery compared to females (67, 132, 133). Conversely, adolescent females (12–18 years old) have a higher number and percentage of circulating CD4^+^ T cells, IgG levels and B cell receptor expression (67, 132, 134). Unfortunately, contradictory findings were reported with respect to CD8^+^ cell counts and CD4^+^/CD8^+^ ratios. Tollerud et al. found males to have higher CD8^+^ cell counts and CD4^+^/CD8^+^ ratio, whereas Bartlett et al. reported the opposite with CD4^+^/CD8^+^ ratio and found no differences in CD8^+^ cell counts (67, 132). The resultant sexual dimorphism in immune response has also been described in bacterial and viral infections with a few exceptions noted in certain cases (128, 135).

Importantly, sexual dimorphism is also apparent in CNS immunity. In an animal model of vesicular stomatitis virus, female mice displayed slower spread of the virus from rostral to caudal ends of the brain, produced lower viral titers, and showed overall enhanced recovery to two different viral strains (136). This last observation showed significant correlation with increased nitric oxide synthases (NOS) production, T cell infiltration, major histocompatibility complex (MHC) class II expression, and a higher frequency of reactive astrocytes (136). In clinical studies, males appear to demonstrate a higher prevalence of certain CNS infection, as demonstrated in bacterial, viral, and TB meningitis (137–139). However, this is not generalized to all CNS infections. For instance, congenital CMV shows equal infection rates across both sexes, but the progression to severe CMV and development of neurological sequelae are twice as common in females than in males (140).

Sex-based differences have also been documented in response to vaccines across the life span. Not only do females elicit stronger antibody responses, but they also experience more adverse reactions following vaccination compared to their male counterparts (141). This has been observed in a variety of vaccines including BCG (142, 143), influenza (144) and measles-mumps-rubella (145). Given the lack of data, the underlying mechanism(s) which mediate sex-based differences in response to vaccines in children are not entirely understood and require further study for application in vaccine design (141).

#### Malnutrition

Malnutrition is a global health problem contributing to more than 40% of deaths among children under the age of five in developing countries (146, 147). Schlaudecker et al., proposed that the relationship between immunity and malnutrition is bi-directional: malnutrition increases the risk of infections and associated mortality, yet infections exacerbate malnutrition through loss of appetite and catabolism induction (148).

Over the years, various associations between innate immune parameters and malnutrition in children have been investigated. In a Zambian cohort of severely malnourished and HIV-infected children, defects in blood DC numbers and functionality were noted (149). Endotoxemia contributed to failed DC maturation (although this occurred in the minority of cases) and low DC numbers. Separately, the DCs of malnourished children without endotoxemia had reduced IL-12 production which improved following nutritional treatment (149). Several clinical and animal studies have reported reduced monocyte/macrophage numbers in malnourished hosts (150–152). The apoptotic marker, CD95, was found to be highly expressed on monocytes in the blood of protein-energy malnourished infants, suggesting that monocytes have a reduced lifespan (150). Despite their reduced numbers, macrophages appear to preserve their microbicidal activity (153, 154). However, impaired chemotaxis, lysosomal enzyme synthesis, microbicidal activity, and glycolytic activity in neutrophils have been noted in malnourished children (155–157). Although moderate and severe malnutrition does not affect the number of circulating NK cells in young children (8–36 months old), their activity was reduced in these patients but normalized following nutritional therapy intervention (158, 159). These alterations in the innate immune system of malnourished children inhibit their first line of defense against disseminated infections such as CNS infections.

Similarly, malnourishment negatively affects the adaptive immune system in young children. Flow cytometric data demonstrated significantly reduced numbers of T cell subsets, particularly CD3^+^, CD4^+^ and CD8^+^, and B cells (CD20^+^) in malnourished children with respiratory and gastrointestinal infections (160). In a separate study comparing the ratios of CD4^+^CD45RO^+^ (memory), CD4^+^CD45RA^+^/CD45RO^−^ (naive) and CD4^+^CD45RA^+^/CD45RO^+^ (Ddull) T cell subsets, malnourished-infected children displayed reduced memory T cells and elevated Ddull T cells compared to well-nourished infected patients (161). Najera et al. also showed that compared to well-nourished infected children, malnourished-infected children had relatively lower circulating numbers of CD4^+^ CD62L^−^ and CD8^+^ CD28^−^ T cells, suggesting T cell functional impairment (162). Apart from the decreased frequencies of certain T cell subsets, a decrease in the production of numerous key cytokines, a marker of decreased functionality, has also been documented. Previous studies investigating peripheral blood mononuclear cells of bacteria-infected malnourished children, reported overexpression of TH2 cytokines (IL-4 and IL-10) and reduced cytokines required for TH1 function (IFN-γ and IL-2) and differentiation (IL-7, 12, 18, 21) (163–165). Additionally, antibody-mediated responses are affected by malnutrition, with increased levels of IgG1 and IgE and reduced B cell numbers; however, TH1 immunoglobulins, IgG2a and IgG3, appear unaffected by malnutrition (160, 166). Thymic atrophy occurs commonly during aging and has also been observed in children with severe acute malnutrition. Consequently, this leads to lower naïve T cell output, reduced adaptive immune responses, and limited TCR diversity (167, 168).

Vaccination in the context of malnutrition has been extensively reviewed in the past (169, 170). Overall, it appears that malnourished children are capable of mounting appropriate responses to a variety of vaccines, with exception of a few studies which noted low seroconversion rates and vaccine-specific antibody titers in severe acutely malnourished children (169, 171, 172). There are several possible explanations to why malnourished children may be more susceptible to infection but mount protective responses to vaccines. Firstly, short-term antibody responses were the focus in these vaccine studies which may have neglected the impact of malnourishment on the longevity and quality of vaccine responses. Secondly, most vaccines are highly adjuvanted allowing them to prevail over the immune impairment caused by malnutrition. Lastly, the morbidity and mortality associated with malnourished-infected children may result from innate immune response impairments, whilst adaptive responses remain sufficient to an extent (172). It is clear that malnourishment, which is prevalent in developing countries where there is also the largest burden of infections, decreases children's innate and adaptive immune response, compounding their risk of severe infections.

#### Maternal Factors

During fetal development, the maternal environment (i.e., maternal nutrition, toxins, infections, stress) is one of the main factors affecting the normal development of the fetus, including its immune system. The fetal and maternal immune systems communicate bidirectionally to ensure maternal immunocompetence and appropriate fetal immune system development (173, 174). Exacerbated maternal immune activation characterized by increases in proinflammatory cytokines can negatively impact the developing immune system of the fetus as well as have neurodevelopmental implications (175). The fetal immune system can be affected differentially across gestational periods, creating “windows” of vulnerability (176).

The fetal immune system is also particularly vulnerable to maternal malnutrition and prenatal stress. Maternal malnutrition leads to changes in placental size, morphology and blood flow which subsequently leads to compromised fetal nutrition and immune system development (177). Moreover, maternal deficiency in micronutrients, such as zinc, has been linked with diminished lymphocyte activity and reduced concentrations of antibodies in infants (178). Both preclinical and clinical research suggest that prenatal stress leads to immune system alterations in infants, of which the latter includes a study examining infectious disease outcomes in children who experienced *in utero* stress (179). The study reported that, compared to non-exposed infants, infants subjected to prenatal stress had a 25 and 31% increased risk of developing severe infections (meningitis, sepsis, ethmoiditis) and less severe infections (pneumonia, bronchitis, upper respiratory tract infections) during childhood respectively (179). In a separate study, infants who experienced maternal prenatal stress exhibited altered innate and adaptive immune responses at 6 months of age (180). For instance, responder frequencies of IFN-γ to antigens were decreased whereas IL-4 was increased suggestive of a dominant TH2 response (180). Certain immune markers in cord blood have also been associated with a variety of different stresses as reported by mothers. For example, stressful deliveries have been shown to influence lymphocyte subsets in neonates (181). Additionally, Wright et al. reported an association between prenatal maternal stress (individual stressors and socioeconomic stress) and alterations in cord blood mononuclear cell cytokine responses (182). Moreover, preclinical studies comparing neonate germ-free mice and conventionally colonized mice demonstrated the importance of maternal microbiota exposure in influencing key neurodevelopmental events (183, 184). For instance, germ-free mice exhibited decreased tumor necrosis factor (TNF)-α and IL-β expression, altered cell death, and an increase in both microglial number and size, compared to conventionally colonized mice (183). The role of microglia during CNS development will be discussed further in part two of this review series.

Several other studies have also demonstrated the importance of microbial colonization on the development of optimal postnatal host immune responses (185–188); as reviewed by (4). Based on these findings, it is evident that prenatal maternal and postnatal environmental factors can shape early life immune responses and neurodevelopmental events, leading to changes that could potentially persist during early childhood. Further research into these factors could have important implications in understanding the state of immunity in neonates, assessing their susceptibility to infectious diseases, and identifying potential interventions that can minimize their risk to infections.

## Bidirectional Crosstalk Between the Peripheral Immune System and CNS

**Figure 3:**
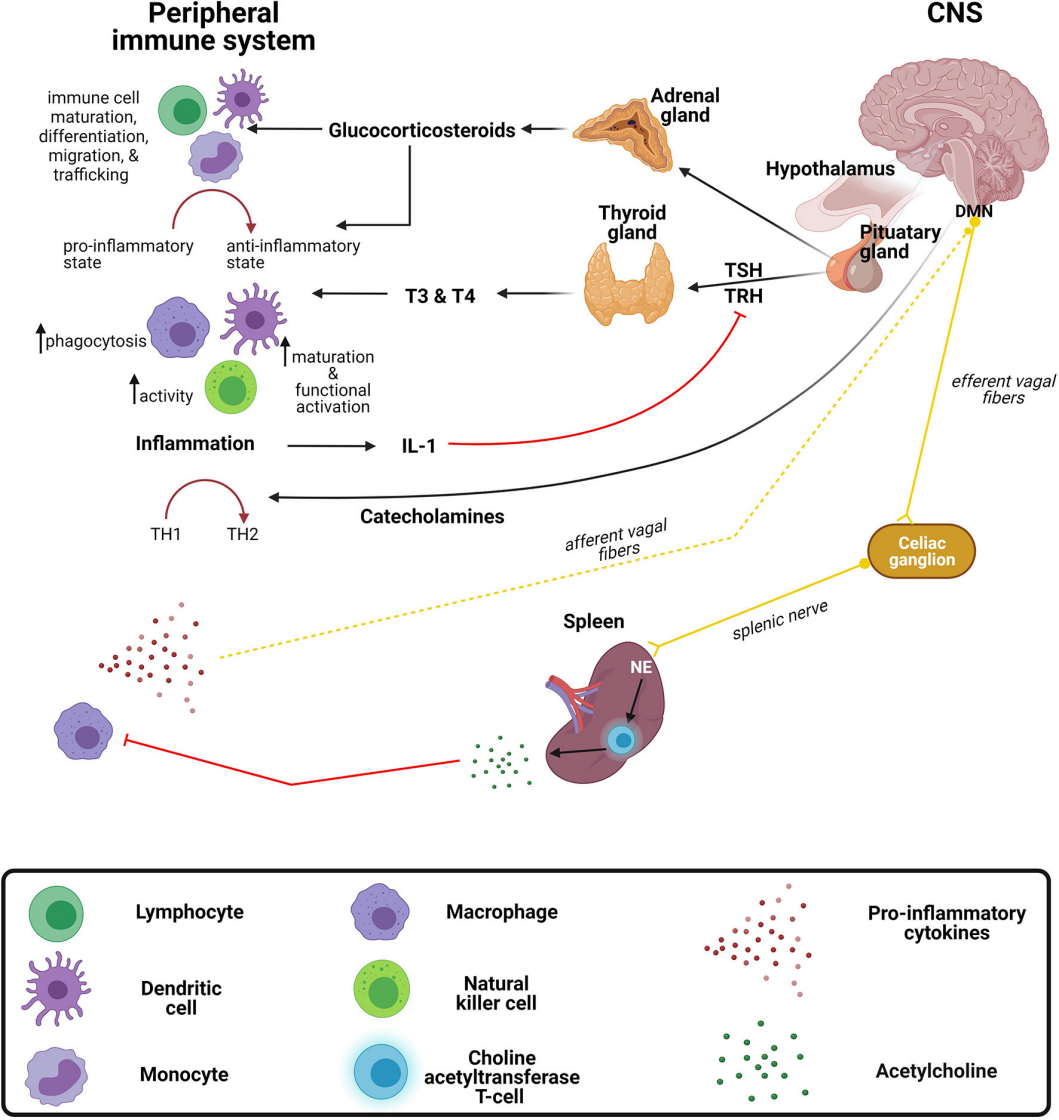
Crosstalk between the peripheral immune system and central nervous system. The brain exerts immunomodulatory effects on the immune system through the release of (1) corticosteroids and catecholamines from the hypothalamic-pituitary adrenal (HPA) axis and (2) thyroid hormones from the hypothalamic-pituitary thyroid (HPT) axis. The peripheral immune system regulates the central nervous system (CNS) *via* the release of inflammatory mediators and signaling *via* the vagus nerve. DMN, dorsal motor nucleus of the vagus nerve; IL-1, interleukin-1; NE, norepinephrine; TH, T-helper; TRH, thyroid-releasing hormone; TSH, thyroid-stimulating hormone; T4, thyroxine; T3, triiodothyronine.

Although the CNS is separated from the peripheral immune system by the semi-permeable blood-brain barrier (BBB), the peripheral immune system and the brain communicate in a bidirectional manner. This bidirectional communication is possible due to the receptors shared between the systems. For instance, peripheral immune cells such as monocytes/macrophages, T and B cells, and DCs express neural receptors including α- and β-adrenergic and acetylcholine receptors (189, 190). Conversely, sensory neurons also express receptors for cytokines such as TNF-α, as well as certain PRRs such as TLR (191). The brain regulates the immune system *via* hormonal pathways, including the hypothalamic-pituitary adrenal (HPA) and hypothalamic-pituitary-thyroid (HPT) axes, and neuronal mechanisms including the autonomic nervous system. Whereas the immune system can regulate the CNS directly by releasing cytokines or indirectly through secondary messengers and signaling *via* the vagus nerve (Figure 3) (192, 193).

Glucocorticoids, a class of corticosteroids which is released upon stimulation of the HPA axis, modulates a variety of immune functions. Many infections are known to activate the HPA axis *via* cytokines that stimulate the hypothalamus to produce corticotropin releasing hormone (CRH) which stimulates the anterior pituitary to excrete adrenocorticotropin hormone (ACTH), culminating with the release of glucocorticoid from the adrenal glands (194). The most characteristic immunomodulatory effect is a shift from a proinflammatory to an anti-inflammatory response (195). This shift toward an anti-inflammatory response is achieved by the suppression of transcription factors, NF-κB and activator protein 1 (AP-1), resulting in the downregulation of genes responsible for encoding proinflammatory mediators (196, 197). In general, glucocorticoids regulate immune cell maturation, differentiation, migration, and trafficking (198, 199). Activation of the HPA axis, such as with stress, increases susceptibility to infectious diseases, such as viral infections and bacterial infections (200). Similar to the immune system, the HPA axis changes during development, with initially low or hyporeactive response to stimuli in infancy (200). Interestingly, a study in rats demonstrated that this hyporeactive response results from immaturity of neural pathways providing stimulatory signals and inhibitory signals (200).

Similar to the HPA axis, the HPT axis also has immunomodulatory effects on certain aspects of the immune system. Thyroid hormones, triiodothyronine (T3), and thyroxine (T4) exert stimulatory effects on immune cells (201–203). For instance, increased DC functional activation and phenotypic maturation (204), increased NK cell activity (205), and increased macrophage-mediated phagocytosis (206) have been observed following the administration of these hormones. Furthermore, during inflammation, the release of cytokines such IL-1, can indirectly inhibit the secretion of thyroid-stimulating hormone (TSH) through the inhibitory effects exerted on thyrotropin-releasing hormone (TRH) (207), often causing the condition known as “euthyroid sick syndrome.”

Catecholamines, such as norepinephrine and adrenaline, are neurotransmitters released from the sympathetic and parasympathetic nerve fibers of the autonomic nervous system regulate the immune system both locally and systemically (208, 209). As with glucocorticoids, catecholamines drive a TH2 shift systemically (210), which has been substantiated by both preclinical and clinical studies (211–213). For instance, administration of either α- or β-adrenergic receptor agonists resulted in reduced LPS-induced TNF-α production in mice (211). Furthermore, the circulating catecholamine, adrenaline, exerts inhibitory effects on NK cells by either acting on α- or β-adrenergic receptors expressed on the cell surface or through IL-12 and IFN-γ suppression, both of which are crucial for NK cell activity (212, 213). The effect of catecholamines on TH1/TH2 balance may not always be generalizable, as under certain conditions they might differentially affect local immune responses (195). The outcome of infectious diseases is dependent on the balance of TH1/TH2 responses. Although it is clear that the above-mentioned hormones and neurotransmitters exert a variety of immunomodulatory effects, the implications on CNS infections require further investigation (214).

Conversely, the immune system can modulate the brain by stimulating the vagus nerve and subsequently activating neural pathways *via* systemically released cytokines (189, 215). The vagus nerve is one of three major nerves originating from the parasympathetic nervous system. It further regulates immune responses to infections and inflammation through a neural circuit known as the inflammatory reflex (216). Briefly, inflammatory mediators released by peripheral immune cells during bacterial and/or viral infections are capable of activating vagal sensory neurons. Following activation of vagal sensory nerve fibers, the signal is propagated to the splenic nerve which in turn triggers the release of norepinephrine from adrenergic receptors, culminating on a specialized subset of T cells that release acetylcholine (189, 216). Acetylcholine subsequently controls the inflammatory response and suppresses the release of proinflammatory cytokines by acting on macrophages (189). Interest in utilizing vagus nerve stimulation to treat chronic inflammatory conditions, such as rheumatoid arthritis, has grown in recent years and there are current pilot studies in humans with promising results. For example, Drewes et al. found that vagus nerve stimulation in patients with rheumatoid arthritis reduced IFN-γ and was well tolerated in those with high disease burden (217). Further studies to investigate application of vagus nerve stimulation in infections may be warranted.

## Conclusion

This review summarizes differences in the immune system across different ages and highlights the various internal and external factors which differentially impact immune development and the subsequent immune responses that ensue. It is well established that immunity varies with age, as early childhood (infants to preschool children) marks a vulnerable period in which the immune system is highly susceptible to infections, not only within the periphery but CNS as well. In contrast, adolescents and adults appear better equipped in responding to infections as the immune system seems to reach full functionality during these periods. However, although the developing immune system is vulnerable, the absence of robust immune responses may also be considered a form of resilience and a means of avoiding a potentially detrimental overload of immune reactions during early life (4, 128). The balance of incremental development of the early immune system and adequate pathogen defense is a tenuous one and is likely overwhelmed in children with a high pathogen exposure and suboptimal environmental circumstances (e.g., maternal stress, malnutrition). Further, communication between the peripheral immune system and CNS occurs through integrative pathways establishing a controlled regulatory system, yet perturbations occurring at any level can alter disease susceptibility and severity (193, 218). Therefore, optimal neuro-immune crosstalk is essential in the wake of infectious insults, especially in young children given their vulnerability. Further insight into the impact of CNS infections on the developing brain will be discussed in part two of this review series.

## Author Contributions

UR and ET conceptualized the manuscript. GS performed the literature search and wrote the initial draft. All authors edited the manuscript. All authors contributed to the article and approved the submitted version.

## Funding

GS is supported by the South African National Research Foundation (NRF) Full Cost of Study Scholarship (980000001087). ET is supported by the NIH NIAID K08AI139371 and the Hartwell Foundation −2018 Individual Biomedical Research Award. UR is supported by the Crick African Network Fellowship: Career Accelerator Award (CAN/C00001/1).

## Conflict of Interest

The authors declare that the research was conducted in the absence of any commercial or financial relationships that could be construed as a potential conflict of interest.

## Publisher's Note

All claims expressed in this article are solely those of the authors and do not necessarily represent those of their affiliated organizations, or those of the publisher, the editors and the reviewers. Any product that may be evaluated in this article, or claim that may be made by its manufacturer, is not guaranteed or endorsed by the publisher.
